# Ginsenoside Rg1 can reverse fatigue behavior in CFS rats by regulating EGFR and affecting Taurine and Mannose 6-phosphate metabolism

**DOI:** 10.3389/fphar.2023.1163638

**Published:** 2023-04-10

**Authors:** Chaofang Lei, Jiaxu Chen, Zhen Huang, Yinian Men, Yue Qian, Mingzhi Yu, Xinyi Xu, Lin Li, Xin Zhao, Youming Jiang, Yueyun Liu

**Affiliations:** ^1^ School of Traditional Chinese Medicine, Beijing University of Chinese Medicine, Beijing, China; ^2^ Guangzhou Key Laboratory of Formula-Pattern of Traditional Chinese Medicine, School of Traditional Chinese Medicine, Jinan University, Guangzhou, China; ^3^ School of Life Sciences, Beijing University of Chinese Medicine, Beijing, China

**Keywords:** ginsenoside Rg1, metabolomics, network pharmacology, EGFR, AKT1, VEGFA, taurine, Mannose 6-phosphate

## Abstract

**Background:** Chronic fatigue syndrome (CFS) is characterized by significant and persistent fatigue. Ginseng is a traditional anti-fatigue Chinese medicine with a long history in Asia, as demonstrated by clinical and experimental studies. Ginsenoside Rg1 is mainly derived from ginseng, and its anti-fatigue metabolic mechanism has not been thoroughly explored.

**Methods:** We performed non-targeted metabolomics of rat serum using LC-MS and multivariate data analysis to identify potential biomarkers and metabolic pathways. In addition, we implemented network pharmacological analysis to reveal the potential target of ginsenoside Rg1 in CFS rats. The expression levels of target proteins were measured by PCR and Western blotting.

**Results:** Metabolomics analysis confirmed metabolic disorders in the serum of CFS rats. Ginsenoside Rg1 can regulate metabolic pathways to reverse metabolic biases in CFS rats. We found a total of 34 biomarkers, including key markers Taurine and Mannose 6-phosphate. AKT1, VEGFA and EGFR were identified as anti-fatigue targets of ginsenoside Rg1 using network pharmacological analysis. Finally, biological analysis showed that ginsenoside Rg1 was able to down-regulate the expression of EGFR.

**Conclusion:** Our results suggest ginsenoside Rg1 has an anti-fatigue effect, impacting the metabolism of Taurine and Mannose 6-phosphate through EGFR regulation. This demonstrates ginsenoside Rg1 is a promising alternative treatment for patients presenting with chronic fatigue syndrome.

## 1 Introduction

Chronic fatigue syndrome (CFS) is a common (0.006%–3%), severely disabling disorder characterized by long-term extreme fatigue that persists even after resting (Nguyen et al., 2019; Moore et al., 2021). CFS is accompanied by depression, concentration difficulty and memory loss (Cvejic et al., 2016; Chaves-Filho et al., 2019). An estimated 1–5 million people present with CFS in Europe every year, with an estimated annual cost of approximately €40 billion in health expenses (Nacul et al., 2021); while in the United States CFS affects 1–2.5 million people yearly and costs between $1.7∼$24 billion (Bested and Marshall, 2015), compared to 44.71 ± 6.10 cases/100,000 people in South Korea (Lim et al., 2021). In China, a previous cross-sectional study found CFS is prevalent among adolescents (Shi et al., 2018).

At present, CFS’s etiology remains unknown, and there are no effective treatments available (Sandler and Lloyd, 2020), with deficient and unspecific diagnostic criteria further preventing appropriate approaches. This makes it imperative to increase the efforts for discovering biomarkers to aid diagnosis and include Chinese medicine has as a potential area for therapy.

Spleen deficiency represents the core pathogenesis of CFS (Geng and Wang, 2012) and can be treated with ginseng, a classic herbal prescription (Ma et al., 2021) that contains ginsenosides as main active components, of which Rg1, a triterpenoid saponin, is the most abundant (Mohanan et al., 2018; Yousuf et al., 2022). In addition, Rg1 presents anti-fatigue effects, as demonstrated by an increase in swimming, fight, and rest times in CFS model rats, which are associated with an increase in serum IgA, IgG, IgM, IFN-β, IFN-γ, T-AOC and Ache (He et al., 2020). Li et al. (2022) found that Panax notoginseng saponin R1 can be efficiently transformed into ginsenoside Rg1 to enhance anti-fatigue effects. CFS is also associated with several metabolic disorders, including energy, amino acids, nucleotides, nitrogen, hormones, lipids and neurotransmitter-related pathways (Armstrong et al., 2014; Nagy-Szakal et al., 2018). However, it remains unclear whether ginsenoside Rg1 can regulate these metabolic disorders in CFS rats.

Traditional Chinese medicine has played an important role in health protection and disease treatment for thousands of years, and is becoming gradually recognized by the international community, as shown by the development of metabolomics, an emerging systems biology technology whose core idea is similar to the holistic approach of traditional Chinese medicine (Wang et al., 2015). Moreover, network analysis explores the associations between drugs, targets, and diseases through network information, whereby combining it with metabolomics can provide insights into the complex interrelationships between biomarkers and disease.

## 2 Materials and methods

### 2.1 Animals, drug administration and sample collection

We purchased 32 male Sprague-Dawley rats from the Beijing Vital River Laboratory Animal Technology Co., Ltd. [animal license No. SCXK (Beijing) 2016–0006]. The rats were kept under controlled environmental conditions (room temperature 22°C ± 2°C, 12-h light/dark cycle) with free access to standard food and water. All experiments were performed according to the EU (Directive 2010/63/EU) ethical guidelines and were approved by the Animal Care and Therapy Ethics Committee of the Beijing University of Chinese Medicine (BUCM-4-2019030402–1036).

After 1 week of adaptive feeding, all rats were randomly divided into four groups (n = 8 per group), including the normal Control group (Control), the model group (CFS), the model + positive control group (CFS+ Oryzanol&VB1), and the model + ginsenoside Rg1 group (CFS + Rg1). Starting from week 3, all groups were given intragastric administration 30 min before modeling every day.

The rats in each group were given continuous gavage for 2 weeks, once a day, and received the following treatments: 1) Control: intragastric administration of double distilled water (10 ml/kg body weight); 2) CFS: intragastric administration of double distilled water (10 ml/kg body weight); 3) CFS+ Oryzanol&VB1 group: gavage glutamine and vitamin B1, (3.15 mg/kg/d, 10 ml/kg body weight) (Xu et al., 2019); 4) CFS + Rg1 group: intragastric administration of ginsenoside Rg1 (50 mg/kg/d, 10 ml/kg body weight) (Feng et al., 2010; Heinrich et al., 2020).

After performing behavioral tests, we anesthetized the animals using isoflurane. The hippocampus and prefrontal cortex were stripped and quickly preserved in liquid nitrogen. Blood was collected through the abdominal aorta and serum was separated by centrifugation (3500 rpm, 10 min, 4°C). Serum and brain tissue were stored at -80°C until analyzed.

### 2.2 Chemicals and reagents

Ginsenoside Rg1 was purchased from Chengdu DeSiTe Bio-Technology Co., Ltd. with the following specifications: 5 g/bottle (HPLC ≥ 98%, CAS No.22427-39-0). Guweisu tablets were purchased from Beijing Zhongxin Pharmaceutical Co., Ltd. with the following specifications: 10mg/tablet (No.H13020683). Vitamin B1 tablets were purchased from Tianjin Feiying Yuchuan Pharmaceutical Co., Ltd. with the following specifications: 10 mg/tablet (No.12020592). D-xylose was purchased from BioRuler.

UPLC Methanol, Acetonitrile, and ultra-pure water were purchased from Fisher Chemical (Fair Lawn, United States). Uplc-grade FormicAcid was purchased from CNW (Shanghai, China). UPLC Grade 2-Propanol was purchased from Merck (Darmstadt, Germany). 2-Chloro-L-Phenylalanine (≥98%) was obtained from Adamas-beta (Shanghai, China).

Bicinchoninic acid (BCA) protein quantitative detection Kit, SDS-PAGE Gel Preparation Kit, Radio-Immunoprecipitation Assay (RIPA) Lysate, and enhanced chemiluminescence (ECL) were purchased from Servicebio (Wuhan, China). GAPDH (GB15002) was purchased from Servicebio (Wuhan, China). AKT1 (ab81283), VEGFA (ab214424), and EGFR (ab52894) were purchased from Abcam (Shanghai, China).

### 2.3 Chronic fatigue syndrome model establishment

**FIGURE 1:**
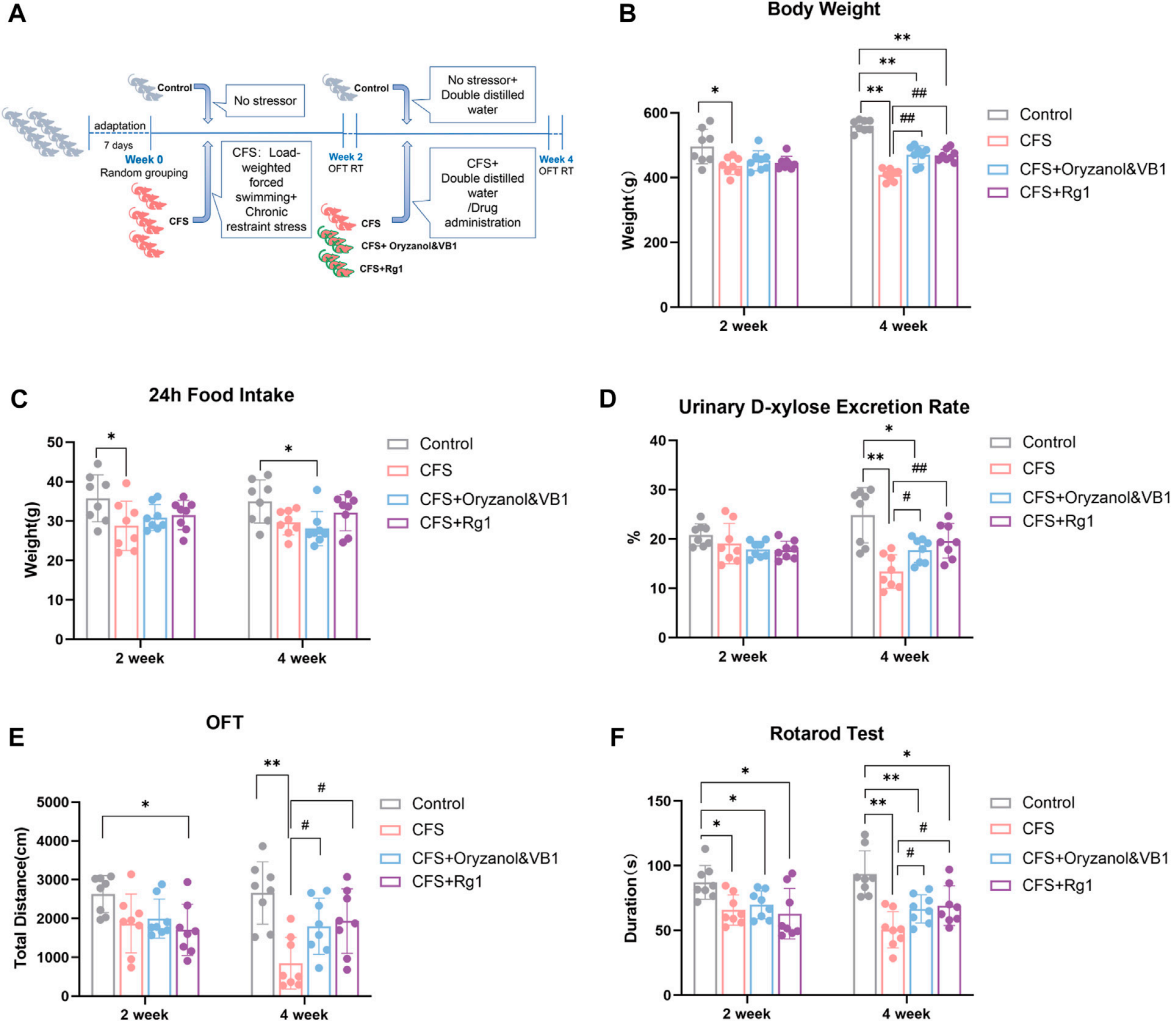
**(A)** Experimental procedure implemented for a CFS rat model. **(B)** Body weight changes in the four rat groups. **(C)** 24-h food intake of the four rat groups. **(D)** 24-h urinary D-xylose excretion rate in the four rat groups. **(E)** Total travel distance of the four rat groups in the Open Field Test. **(F)** Time spent on the rod in the four rat groups (Rotarod Test). Data are shown as the mean ± standard deviation, n = 8. *p < 0.05, **p < 0.01 versus control group; #*p* < 0.05, ##*p* < 0.01 versus CFS group.

A multi-factor modeling method was used to simulate CFS pathogenesis (Shao et al., 2017): 1) Load-weighted forced swimming: Rats in the modeling group swam in a transparent toughened plastic bucket (diameter 20 × height 50 cm) for 10 min every day for 28 days. The water temperature of the swimming pool was controlled at 23°C ± 2°C. A small artery clip was attached to the lead wire about 5% of the body weight of the rat, and then the weight was placed on the back hair of each rat. 2) Restriction: The rats in the model group were bound to the wooden restriction frame (22 × 10 × 2 cm in length and 10 × 2 cm in thickness) for 3 h every day, respectively, and subjected to chronic restriction stress for 4 weeks. The Control group did not participate in the above two modeling methods and was fed normally. The experimental procedure is shown in Figure 1A.

### 2.4 Evaluation of the CFS model

Body weight, 24-h food intake, urine D-xylose and behavioral tests (including the open field and rotarod tests) were performed 2 and 4 weeks after modeling for evaluation. In the open field test, the EthoVision 3.0 behavioral device of the Noldus Company (Netherlands) was used to analyze videos and calculate the 5-min total distance of spontaneous movement of each rat group. A rat rotating rod fatigue instrument (Anhui Zhenghua Biological Instrument Equipment Co., Ltd. ZH-300B) was used to measure the time during which rats were able to sustain a constant 30rpm speed. The upper limit of the recording time was set to 5 min.

### 2.5 LC-MS-based serum metabolomics

#### 2.5.1 Sample preparation

100 μL of serum samples were collected in a 2 ml centrifuge tube. The metabolites were extracted from 400 μL of extract solution (methanol: water = 4:1 (v:v)) containing 0.02 mg/ml internal standard (L-2-chlorophenyl alanine). The sample preparation process is described in a previous study (Lei et al., 2022). Equal amounts of the mixture were extracted from all samples and used as QC samples for LC-MS.

#### 2.5.2 Untargeted LC-MS analysis

We used Thermo’s ultra-high-performance liquid chromatography-tandem time-of-flight mass spectrometry UHPLC-Q Exactive HF-X system as the instrument platform. A total of 2 μL samples were separated using an HSS T3 chromatographic column (100 mm × 2.1 mm i. d. 1.8 µm) and then detected by mass spectrometry. The mobile phase A consisted of 95% water and 5% acetonitrile (containing 0.1% formic acid). The mobile phase B consisted of 47.5% acetonitrile, 47.5% isopropyl alcohol, and 5% water (containing 0.1% formic acid). The column temperature was set to 40 °C. The positive and negative ion scanning modes were used for mass spectrum signal acquisition.

#### 2.5.3 Data processing

LC-MS raw data was imported into Progenesis QI (Waters Corporation, Milford, United States) for baseline filtering, peak identification, integration, retention time correction, and peak alignment. This allowed us to obtain a data matrix of the retention time, mass/charge ratio, and peak intensity. We normalized the data matrix for subsequent analysis. The metabolite information was obtained by matching MS and MS/MS mass spectrometry information with the metabolic public databases HMDB (https://hmdb.ca/) and Metlin (https://metlin.scripps.edu/).

#### 2.5.4 Metabolomic data analysis

The pre-processed data was uploaded to the Majorbio Cloud Platform (http://www.majorbio.com/) for data analysis. The R software package ropls (Version 1.6.2) was used for performing principal component analysis (PCA) and orthogonal least partial square discriminant analysis (OPLS-DA). The stability of the model was evaluated using seven cyclic interaction validations. We also performed a Student’s t-test analysis. The selection of differential metabolites was determined based on the variable weight value (VIP) obtained by the OPLS-DA model and the *p*-value of the student’s t-test. Metabolites with VIP > 1.0 and *p* < 0.05 were selected as metabolic markers. We use the KEGG database (https://www.kegg.jp/kegg/pathway.html) for uncovering metabolic pathways and identify differences in associated metabolites, with *p* < 0.05 used as the standard. The Python software package scipy. stats was used for pathway enrichment analysis, and the most relevant biological pathways were extracted using Fisher’s exact test.

### 2.6 Network analysis

#### 2.6.1 Target prediction

The BATMAN-TCM (http://bionet.ncpsb.org/batman-tcm/) and SwissTargetPrediction (http://www.swisstargetprediction.ch/) databases were used to obtain information on the targets of ginsenoside Rg1.

We identified potential targets by searching CFS keywords on public databases, including Genecards (http://www.genecards.org/), OMIM (https://www.omim.org/), and DRUGBANK (https://go.drugbank.com/).

Differential metabolites were entered into the MetScape database to identify target proteins interacting with these metabolites.

The targets associated with CFS were standardized through the UniProt knowledge Base (http://www.uniprot.org/), converting all retrieved targets into official genetic symbols to facilitate downstream data analysis.

A Venn diagram was drawn using DeepVenn (http://www.deepvenn.com/) to show the common targets of ginsenoside Rg1, the differential metabolites, and CFS-related targets. These genes were considered as potential targets of ginsenoside Rg1 for the treatment of CFS.

#### 2.6.2 Network construction and enrichment analysis

A large number of studies showed proteins exert biological activity through protein-protein interactions. We submitted potential target genes to the Interaction Gene Retrieval Tool (http://string-db.org/) and performed Protein-Protein Interaction Network (PPI) analysis. PPI networks were used to analyze core targets of ginsenoside Rg1 in the CFS-treatment using Cytoscape 3.7.1.

We used OmicShare Tools (https://www.omicshare.com/tools/) for GO and KEGG enrichment analyses to obtain the biological functions and related pathways of ginsenoside Rg1 as potential targets for CFS treatment. The correlation pathways with *p* < 0.05 were considered as statistically significant.

### 2.7 Experimental validation

#### 2.7.1 Quantitative real-time PCR

Total RNA in the hippocampus (Hip) and prefrontal cortex (PFC) was extracted with an RNA extraction solution (Servicebio, China). Reverse transcription was performed with a first-strand cDNA synthesis kit (Servicebio, China) following manufacturer’s guidelines. The expression levels were determined using real-time PCR with SYBR Green Mix (Servicebio, China). Relative mRNA expression was calculated using the 2^−ΔΔCT^ method. GAPDH was used as a normalization control for mRNA levels. The primer sequences are shown in Table 1.

**TABLE 1:** List of PCR primers.

Primers	Forward	Reverse	Gene accession no.
AKT1	CGA​CGT​AGC​CAT​TGT​GAA​GGA​G	ATT​GTG​CCA​CTG​AGA​AGT​TGT​TG	NM_033230.2
VEGFA	GCA​ATG​ATG​AAG​CCC​TGG​AGT	GGC​TTT​GTT​CTA​TCT​TTC​TTT​GGT​C	NM_031836.3
EGFR	AGA​ACA​ACA​CCC​TGG​TCT​GGA​A	CCA​CCA​CTA​CTA​TGA​AGA​GGA​GGC	NM_031507.1
GAPDH	CTG​GAG​AAA​CCT​GCC​AAG​TAT​G	GGT​GGA​AGA​ATG​GGA​GTT​GCT	NM_017008.4

#### 2.7.2 Western blot

RIPA lysate was added to the tissues, crushed by ultrasound, and centrifuged at room temperature for 10 min (12,000 rpm, 4°C). After this, we collected the supernatant, which was considered as the total protein solution. The BCA kit was used to measure the concentration of the protein solution. We added 5× protein loading buffer and denatured the protein in a boiling water bath for 5 min. The same amount of protein solution was subjected to sodium dodecyl sulfate-polyacrylamide gel electrophoresis (SDS-PAGE), after which the gel was transferred to the polyvinylidene fluoride (PVDF) membrane. A 5% skim milk solution containing TBS + Tween (TBST) was kept at room temperature for 30 min and added to the primary antibody at 4°C for overnight incubation in a shak (GAPDH (1:2000), AKT1 (1:1000), VEGFA (1:1000), EGFR (1:1000)). After rinsing with phosphate buffered solution tween (PBST) for 3 × 5 min, the membrane was placed in an horseradish peroxidase (HRP) labeled secondary antibody (1:5000) and incubated at room temperature for 1 h. After rinsing with PBST for 3 × 5 min, the gel imaging system was used for image acquisition. The Alpha Innotech software was used to calculate and analyze the gray scale of protein bands.

### 2.8 Statistical analysis

Statistical analysis was performed using SPSS (version 20.0) and GraphPad Prism (version 9.0). All data are expressed as the mean ± standard deviation ( $\bar{x}\pm SD$ ). Based on data normality and homogeneity of variance, a one-way ANOVA or a non-parametric test were used for comparison. *p* < 0.05 was considered as statistically significant and *p* < 0.01 was considered highly significant.

## 3 Results

### 3.1 Effects of CFS modeling on body weight, food intake, and urinary D-xylose excretion rate in rats

#### 3.1.1 Weight

The body weight of rats in each group is shown in Figure 1B. After 2 weeks of modeling, the rats in the CFS group had lower weight compared to the Control group (*p* = 0.0443). There were no significant differences among model groups. After 4 weeks of modeling, the body weight of the three modeling groups decreased significantly compared with the Control group (*p* < 0.0001). Finally, rat weight in both Oryzanol&VB1 and Rg1 groups recovered significantly compared to the CFS group (*p* = 0.0005, *p* < 0.0001).

#### 3.1.2 Food intake

The 24-h food intake of each group is shown in Figure 1C. After 2 weeks, the food intake of rats in the CFS group was lower than in the Control group (*p* = 0.0237). We found no significant differences among the model groups. After 4 weeks, food intake decreased in the Oryzanol&VB1 group compared to Control (*p* = 0.0240). No significant differences were found between the CFS, the Oryzanol&VB-1 and the Rg1 groups.

#### 3.1.3 Urinary D-xylose excretion rate

The 24-h urinary D-xylose excretion rate of rats in each group is shown in Figure 1D. We found no differences between groups after 2 weeks. However, after 4 weeks of modelling, the urinary D-xylose-excretion rate in the CFS and Oryzanol&VB-1 groups was significantly decreased compared to the Control group (*p* = 0.0011, *p* = 0.0228), and a significant recovery occurred in the Oryzanol&VB1 and Rg1 groups compared to CFS (*p* = 0.0230, *p* = 0.0054).

These results show that CFS modeling produces significant effects on body weight and the urinary D-xylose excretion rate of rats, but no significant differences in food intake. We also note that body weight and urinary D-xylose excretion rate were further decreased in the CFS group over the course of the last 2 weeks of treatment. Crucially, ginsenoside Rg1 intervention could significantly reverse this course, suggesting it is able to improve gastrointestinal absorption.

### 3.2 CFS modeling, spontaneous activity and rotarod test

#### 3.2.1 Open field test

The 5-min total movement distance of each group is shown in Figure 1E. Rats in the Rg1 group moved less than those in the Control group after 2 weeks (*p* = 0.0187), but there were no significant differences among model groups. After 4 weeks, the total movement distance of rats in the CFS group was significantly decreased compared to the Control group (*p* = 0.0007), with a recovery observed in the Oryzanol&VB1 and Rg1 groups (*p* = 0.0296, *p* = 0.0229).

#### 3.2.2 Rotarod test

The time spent on the rod is shown in Figure 1F. Duration decreased in all model groups after 2 weeks (*p* = 0.0105, *p* = 0.0306, *p* = 0.0323), with no significant differences among them. These differences to the Control group became significant after 4 weeks (*p* = 0.0004, *p* = 0.0097, *p* = 0.0286), with both Oryzanol&VB1 and Rg1 groups recovering (*p* = 0.0421, *p* = 0.0440).

These results show CFS modeling had a significant effect on rat spontaneous activity and muscle fatigue. Over the course of the last 2 weeks of modeling, the groups treated with ginsenoside Rg1 recovered significantly, showing its significant anti-fatigue effect.

### 3.3 Metabolomics analysis of serum samples

**FIGURE 2:**
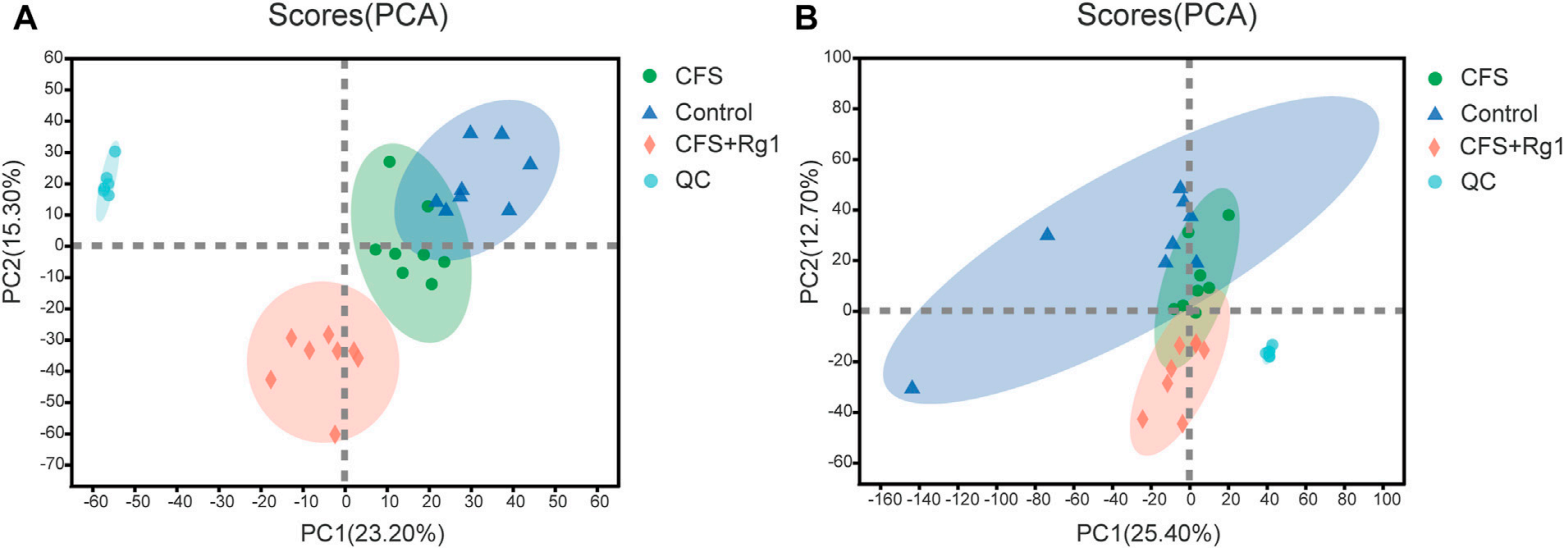
PCA scores for Control (blue), CFS (green) and CFS + Rg1 (pink) groups in positive and negative ion modes **(A, B)**, n = 8.

In order to study changes in endogenous serum metabolites in CFS rats and reveal the mechanisms of ginsenoside Rg1-mediated treatment, we comprehensively scanned serum metabolites of Control, CFS and CFS + Rg1 groups using the UHPLC-QExactiveHF-X system. Principal component analysis (PCA) showed significant differences between these groups. QC analysis showed obvious sample clustering, indicating the analytical method is stable and repeatable. These results are illustrated in Figure 2.

**FIGURE 3:**
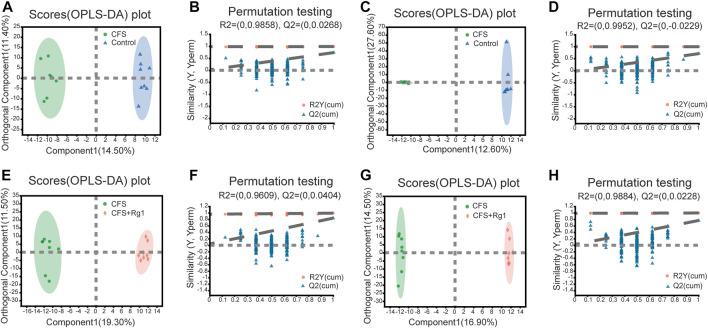
OPLS-DA and permutation tests in the positive **(A, B)** and negative **(C, D)** ion modes of Control and CFS groups, n = 8. OPLS-DA and permutation tests in positive **(E, F)** and negative **(G, H)** ion modes in CFS and CFS + Rg1 groups, n = 8.

OPLS-DA was further used to screen for potential biomarkers. As shown in Figures 3A–D, samples from the Control and the CFS groups were significantly different. The R2X, R2Y and Q2 values of permutation tests applied in the positive ion mode were 0.259, 0.99 and 0.739, respectively; and 0.567, 0.998 and 0.709, respectively, in the negative ion mode. The CFS and the CFS + Rg1 groups were also clearly different. In these groups, the R2X, R2Y and Q2 of permutation tests in the positive ion mode were 0.308, 0.993 and 0.84, respectively; and 0.383, 0.999 and 0.766, respectively, in the negative ion mode. These results are shown in Figures 3E–H and indicate the model has good explanatory and predictive abilities, with no overfitting present.

**FIGURE 4:**
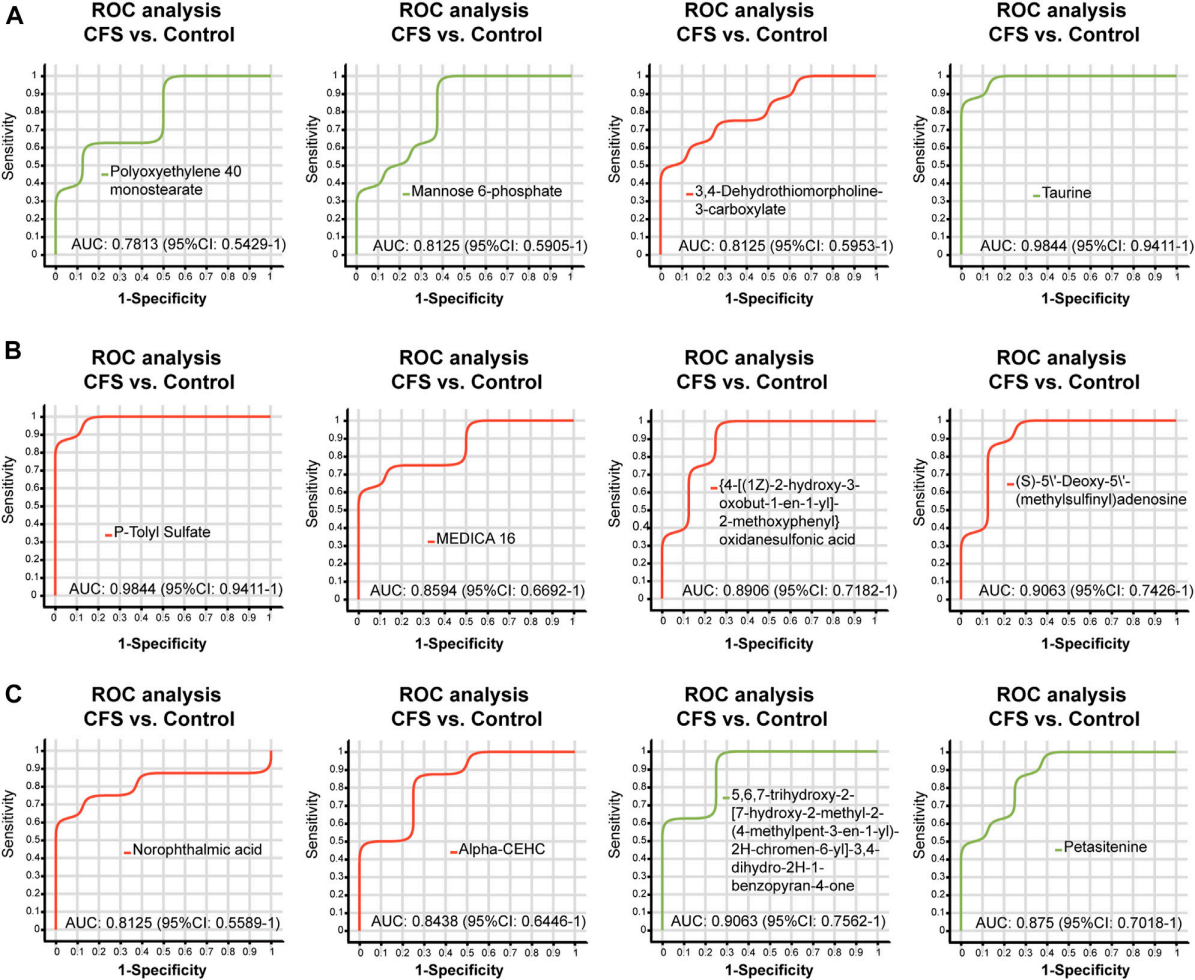
ROC curve analysis of differential metabolites **(A–C)** regulated by ginsenoside Rg1 (CFS vs. Control). The closer the AUC is to 1, the better the diagnostic prediction. AUC has low accuracy between 0.5 and 0.7, and high accuracy above 0.7.

**FIGURE 5:**
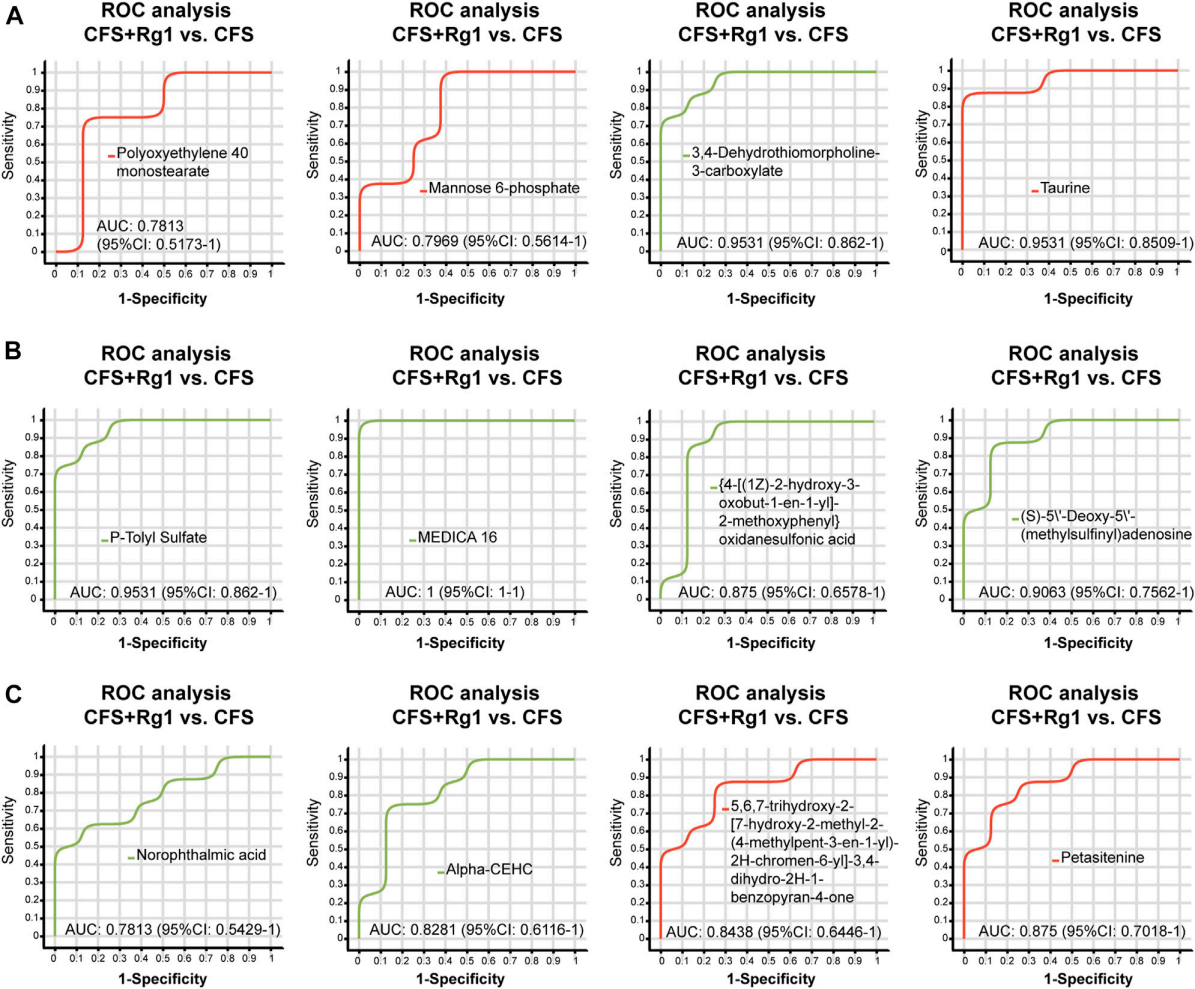
ROC curve analysis of differential metabolites **(A–C)** regulated by ginsenoside Rg1 (CFS + Rg1 vs. CFS). The closer the AUC is to 1, the better the diagnostic prediction. AUC has low accuracy between 0.5 and 0.7, and high accuracy above 0.7.

We found a total of 34 differential metabolites that are potential biomarkers using the screening conditions VIP > 1.0 and *p* < 0.05 (Table 2). Of these, 12 and 22 metabolites were increased and decreased, respectively, in the CFS group compared to Control. Ginsenoside Rg1 treatment could restore Polyoxyethylene 40 monostearate, Mannose 6-phosphate, Taurine, Petasitenine and 5,6,7-trihydroxy-2-[7-hydroxy-2-methyl-2-(4-methylpent-3-en-1-yl)-2H-chromen-6-yl]-3,4-dihydro-2H-1-benzopyran-4-one levels in CFS rats, while lowering 3,4-Dehydrothiomorpholine-3-carboxylate, P-Tolyl Sulfate, MEDICA 16, {4-[(1Z)-2-hydroxy-3-oxobut-1-en-1-yl]-2-methoxyphenyl}oxidanesulfonic acid, (S)-5′-Deoxy-5'-(methylsulfinyl)adenosine, Norophthalmic acid, and Alpha-CEHC. ROC curve analysis showed good diagnostic effect for these metabolites (AUC > 0.7; Figure 4, Figure 5).

**TABLE 2:** Differential metabolites associated with ginsenoside Rg1 in serum.

NO.	Metabolite	M/Z	Formula	Trend CFS/Control	Trend CFS + Rg1/CFS
1	PC(18:0/0:0)	524.3705732	C26H54NO7P	↓**	↓^##^
2	3-Pyridinebutanoic acid	331.1647303	C9H11NO2	↓**	↓^#^
3	PC(17:0/0:0)	510.3549983	C25H52NO7P	↓**	↓^##^
4	N6-Methyl-2′-deoxyadenosine	266.1231266	C11H15N5O3	↓**	↓^#^
5	Blepharin	310.0951397	C14H17NO8	↓*	↓^#^
6	N-Succinyl-2-amino-6-ketopimelate	353.0929004	C11H15NO8	↓**	↓^#^
7	5-Methoxytryptophan	217.0970874	C12H14N2O3	↑*	↑^##^
8	Polyoxyethylene 40 monostearate	346.3312399	C20H40O3	↓*	↑^#^
9	Azaspiracid	443.7371548	C47H71NO12	↑**	↑^##^
10	Palmitoyl-L-carnitine	400.3418928	C23H45NO4	↓*	↓^##^
11	S-(PGJ2)-glutathione	624.2952782	C30H47N3O10S	↑**	↑^#^
12	PC(20:4/0:0)	566.3210231	C28H50NO7P	↓*	↓^##^
13	Methyl cellulose	487.2790688	C20H38O11	↓**	↓^##^
14	Octadecenoylcarnitine	426.3574449	C25H47NO4	↓**	↓^##^
15	Tanacetol B	314.2321816	C17H28O4	↓**	↓^#^
16	Tyrosyl-Serine	233.0919633	C12H16N2O5	↑*	↑^##^
17	Arginyl-Proline	316.1363843	C11H21N5O3	↓*	↓^#^
18	Mannose 6-phosphate	261.0393069	C6H13O9P	↓*	↑^#^
19	Maysin 3′-methyl ether	307.082906	C28H30O14	↓*	↓^##^
20	3,4-Dehydrothiomorpholine-3-carboxylate	146.0269537	C5H7NO2S	↑*	↓^##^
21	Taurine	126.0220252	C2H7NO3S	↓**	↑^##^
22	Norophthalmic acid	274.1044428	C10H17N3O6	↑*	↓^#^
23	L-Aspartic Acid	132.0293516	C4H7NO4	↓**	↓^#^
24	3-Hydroxy-L-proline	307.1145828	C5H9NO3	↓**	↓^#^
25	Acetyl-DL-Leucine	172.0972364	C8H15NO3	↓**	↓^##^
26	P-Tolyl Sulfate	187.0064671	C7H8O4S	↑**	↓^##^
27	Alpha-CEHC	313.1194597	C16H22O4	↑*	↓^#^
28	MEDICA 16	341.2695802	C20H38O4	↑*	↓^##^
29	5,6,7-trihydroxy-2-[7-hydroxy-2-methyl-2-(4-methylpent-3-en-1-yl)-2H-chromen-6-yl]-3,4-dihydro-2H-1-benzopyran-4-one	419.1530259	C25H26O7	↓**	↑^#^
30	Petasitenine	402.1502652	C19H27NO7	↓**	↑^##^
31	Furanogermenone	267.1137544	C15H20O2	↑**	↑^#^
32	{4-[(1Z)-2-hydroxy-3-oxobut-1-en-1-yl]-2-methoxyphenyl}oxidanesulfonic acid	269.0126903	C11H12O7S	↑**	↓^#^
33	(S)-5′-Deoxy-5'-(methylsulfinyl)adenosine	294.0653396	C11H15N5O4S	↑**	↓^##^
34	LysoPC(18:0)	558.3330623	C26H54NO7P	↓**	↓^##^

↑ represents upregulation and ↓ represents downregulation.

**p < 0.01, *p < 0.05 as CFS group versus control group.

^##^ *p* < 0.01, ^#^ *p* < 0.05 as CFS + Rg1 group versus CFS group.

### 3.4 Potential biomarker identification and metabolic pathway analysis

**FIGURE 6:**
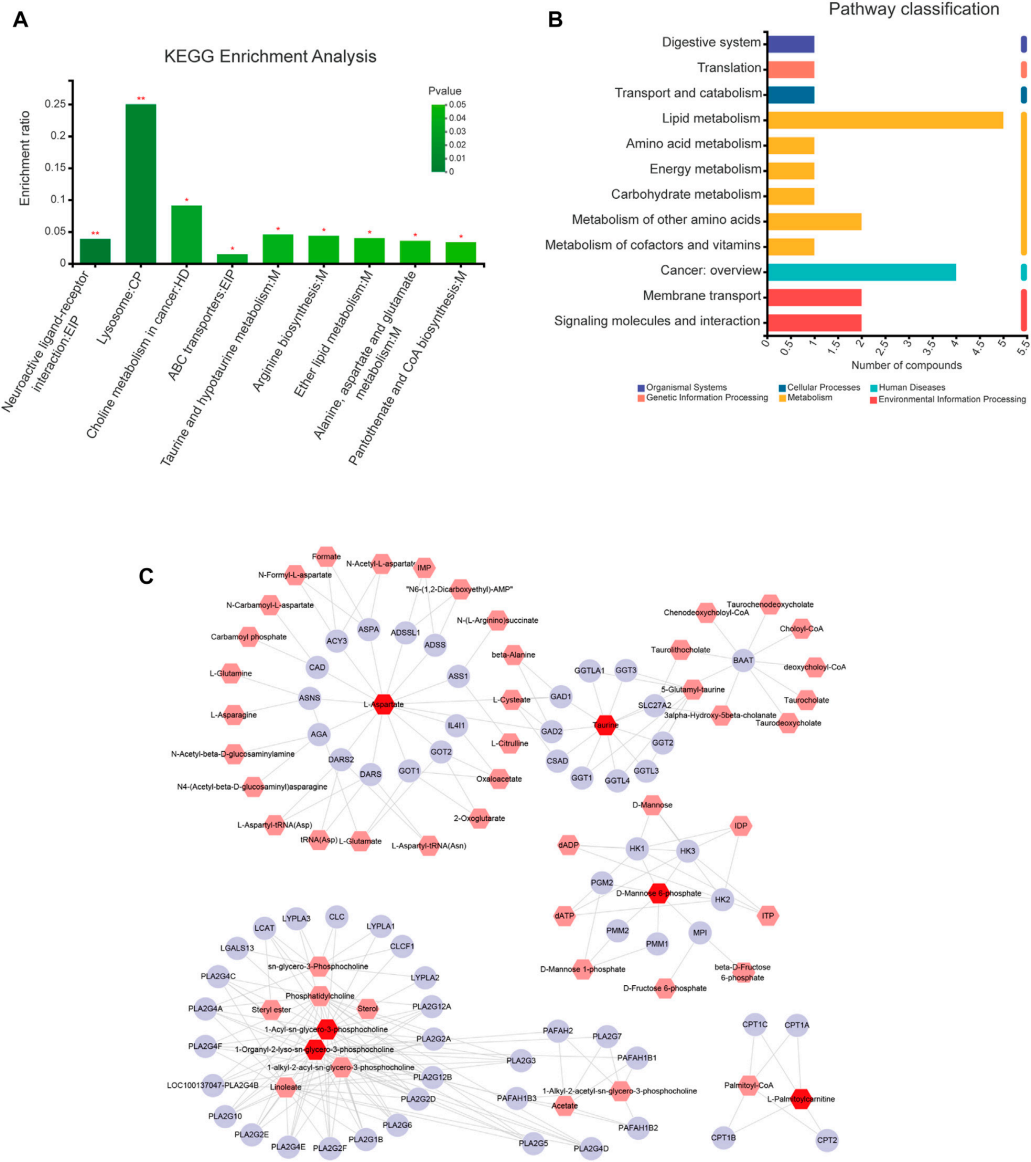
Pathway and network analyses of differential metabolites. **(A)** KEGG enrichment analysis of differential metabolites. **(B)** KEGG classification analysis of differential metabolites. **(C)** “Metabolite-gene” network for anti-fatigue effects of ginsenoside Rg1. Red, pink, and purple nodes represent differential metabolites, interacting metabolites, and interacting proteins, respectively. The edges represent biochemical reactions.

KEGG pathway enrichment analysis of differential metabolites was performed using the Majorbio Cloud Platform. We identified nine pathways using *p* < 0.05 (Figure 6A) associated with the metabolites L-Aspartic Acid, Taurine, Mannose 6-phosphate, LysoPC(18:0), PC(17:0/0:0), and PC(18:0/0:0). Five of these pathways are particularly associated with ginsenoside Rg1 treatment of CFS, including *Taurine and hypotaurine metabolism; Arginine biosynthesis; Ether lipid metabolism; Alanine, aspartate and glutamate metabolism;* and *Pantothenate and CoA biosynthesis*. The Lipid metabolism category included the highest number of differential metabolites (Figure 6B).

The differential metabolites were imported into the MetScape database to construct a “Compound Gene” network (Figure 6C) consisting of 64 target proteins. The related differential metabolites included were L-Aspartic Acid, Taurine, Mannose 6-phosphate, LysoPC(18:0) (1-Organyl-2-lyso-sn-glycero-3-phosphocholine), PC(17:0/0:0) (1-Acyl-sn-glycero-3-phosphocholine), and Palmitoyl-L-carnitine. Our results demonstrate that Taurine and Mannose 6-phosphate are key metabolic markers of ginsenoside Rg1 treatment of CFS.

### 3.5 Network analysis

#### 3.5.1 Ginsenoside Rg1 acts on potential CFS targets

A total of 107 ginsenoside Rg1 targets were obtained through the BATMAN-TCM and SwissTargetPrediction databases. In addition, a total of 3796 disease targets of CFS were obtained from the Genecards, OMIM, and DRUGBANK databases. The differential metabolites were imported into the MetScape database to construct a “Compound-Gene” network, allowing us to identify a total of 64 target proteins (Figure 6C).

**FIGURE 7:**
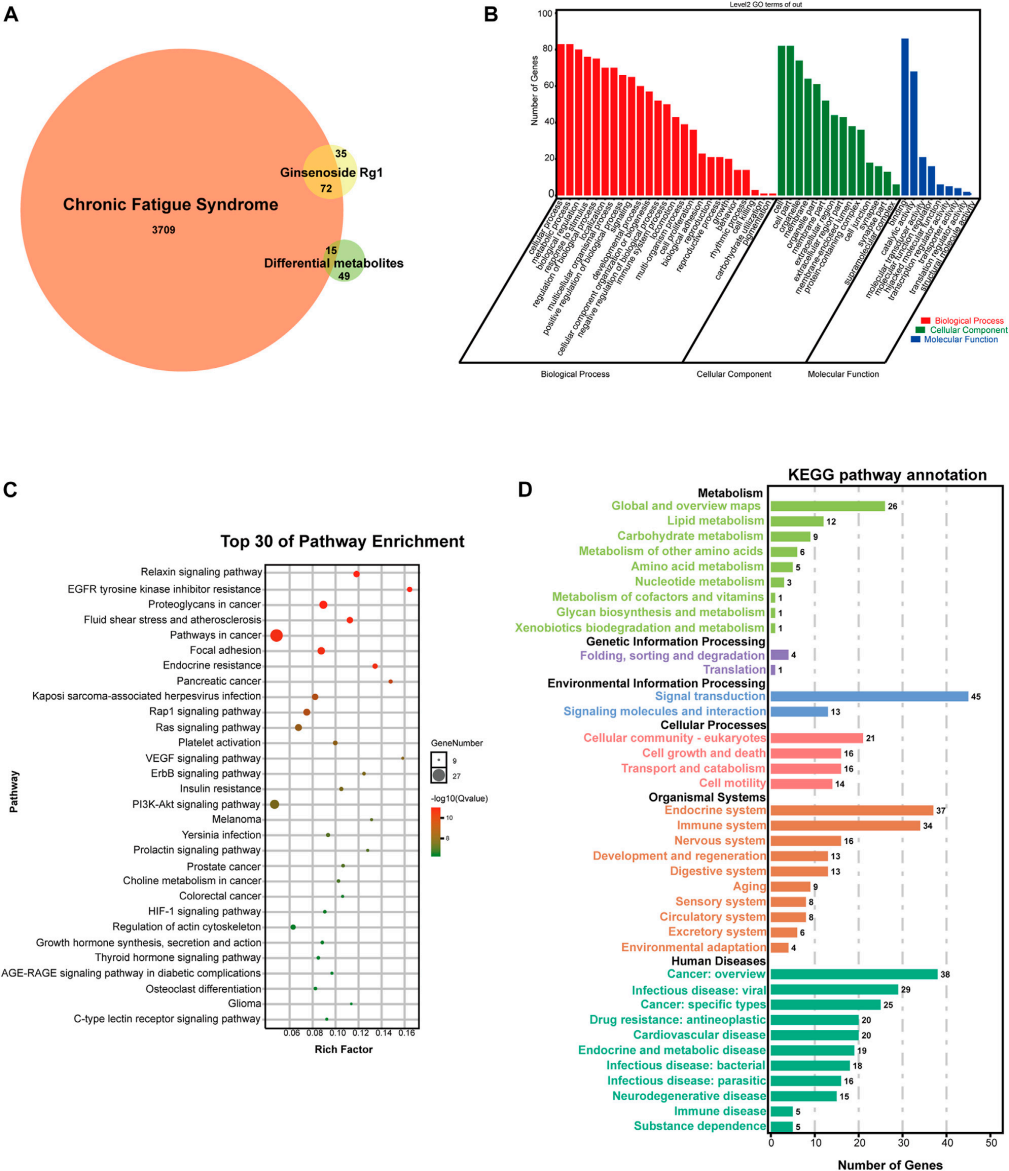
Network analysis of potential targets. **(A)** VENN diagram of potential targets. **(B)** GO analysis of potential targets. **(C)** KEGG pathway enrichment analysis of potential targets. **(D)** KEGG pathway annotation of potential targets.

There were 72 identical targets of CFS and ginsenoside Rg1, and 15 identical targets of CFS and differential metabolites, totaling 87 potential ginsenoside Rg1 targets for CFS treatment (Figure 7A).

#### 3.5.2 Ginsenoside Rg1 pathways of action

We used the OmicShare cloud platform to perform GO enrichment analysis and elucidate the biological associations of potential targets. We identified a total of 48 GO terms for ginsenoside Rg1, 25 for biological processes (BP), 14 for molecular functions (MF), and nine for cellular components (CC) (Figure 7B). BP results predicted the involvement of a high number of genes in cellular process, metabolic process, biological regulation, response to stimulus, localization, multicellular organismal process, positive developmental process, negative regulation of biological process, immune system process, locomotion, multi-organism proces, cell proliferation, and biological adhesion. In addition, CC and MF results revealed that the anti-fatigue effects of ginsenoside Rg1 were mainly associated with cell, organelle, membrane, extracellular region, membrane-enclosed lumen, protein-containing complex, cell junction, synapse, supramolecular complex, catalytic activity, molecular transducer activity, molecular function regulator, hijacked molecular function, transcription regulator activity.

We also performed KEGG analysis to evaluate the functional pathways associated with ginsenoside Rg1 treatment of CFS. Figure 7C shows the top 30 signaling pathways obtained from the KEGG enrichment, while Figure 7D shows KEGG pathway annotation. The main therapeutic pathways associated with ginsenoside Rg1 treatment are EGFR tyrosine kinase inhibitor resistance, Relaxin signaling pathway, Endocrine resistance, Rap1 signaling pathway, Ras signaling pathway, VEGF signaling pathway, ErbB signaling pathway, PI3K-Akt signaling pathway, Prolactin signaling pathway, HIF-1 signaling pathway. An annotated statistical diagram of these pathways revealed ginsenoside Rg1 anti-fatigue effect impacts the Immune system, Nervous system, Endocrine system, Lipid metabolism, Amino acid metabolism, Metabolism of cofactors and vitamins.

#### 3.5.3 PPI network analysis of ginsenoside Rg1’s anti-fatigue effects

**FIGURE 8:**
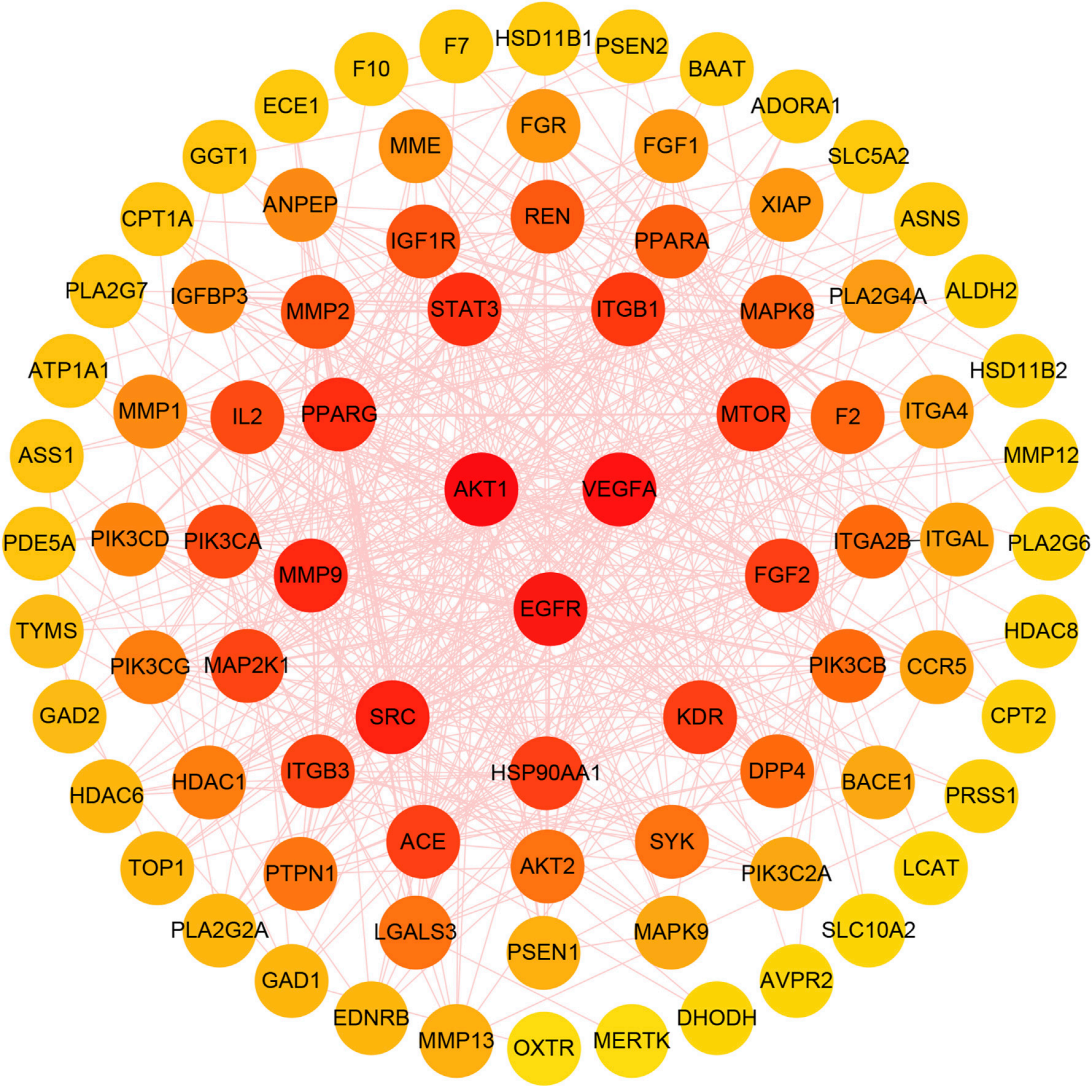
The gradual change of circle colors from red to yellow represents the change in Degree values, from large to small. The three targets in the center of the figure have the highest Degree values.

The protein-protein interaction relationship of potential targets was obtained through the STRING database and consisted of a PPI network containing 87 nodes and 587 edges. This information was imported into CytoScape3.7.1 software to visualize network relationships. The CytoHubba function was used to calculate the Degree. The results show that the top three targets were AKT1, VEGFA and EGFR, with degrees of 49, 44 and 43, respectively. These targets were therefore considered as key anti-fatigue targets of ginsenoside Rg1 (Figure 8).

### 3.6 Effects of ginsenoside Rg1 treatment on mRNA expression of AKT1, VEGFA and EGFR

**FIGURE 9:**
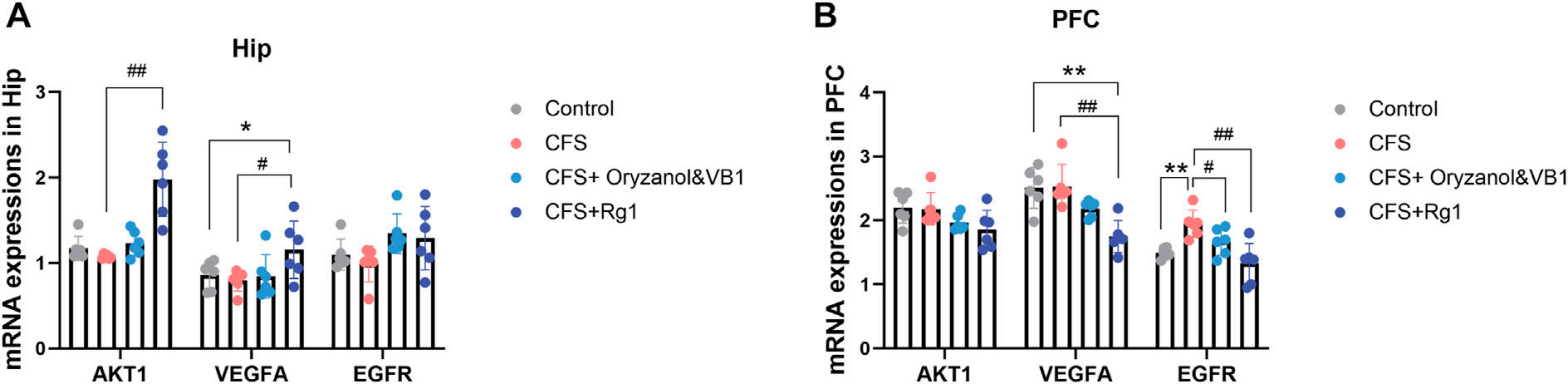
**(A)** Relative mRNA expression of AKT1, VEGFA and EGFR in the hippocampus (Hip) after 2 weeks of treatment. **(B)** Relative mRNA expression of AKT1, VEGFA and EGFR in the prefrontal cortex (PFC) after 2 weeks of treatment. Data are mean ± standard deviation, n = 6. *p < 0.05, **p < 0.01 versus Control group; #*p* < 0.05, ##*p* < 0.01 versus CFS group.

The mRNA expressions of AKT1, VEGFA and EGFR in the rat hippocampus and prefrontal cortex are shown in Figures 9A,B. In the hippocampus, VEGFA expression was increased in the CFS + Rg1 group compared to the Control group (*p* = 0.043), while the CFS + Rg1 showed increased expressions of AKT1 and VEGFA compared to CFS (*p* = 0.001, *p* = 0.015). The expression of EGFR remained relatively constant. As for the prefrontal cortex, VEGFA expression was significantly decreased in CFS + Rg1 compared to Control (*p* = 0.005), and EGFR expression was significantly increased in CFS (*p* = 0.002). In addition, VEGFA and EGFR levels were significantly lower in the CFS+ Rg1 group compared with the CFS group (*p* = 0.006, *p* < 0.001) and EGFR levels were lower in the CFS+ Oryzanol&VB1 group (*p* = 0.038). These results show ginsenoside Rg1 regulates EGFR in the prefrontal cortex of CFS rats.

### 3.7 Protein expression levels of AKT1, VEGFA and EGFR

**FIGURE 10:**
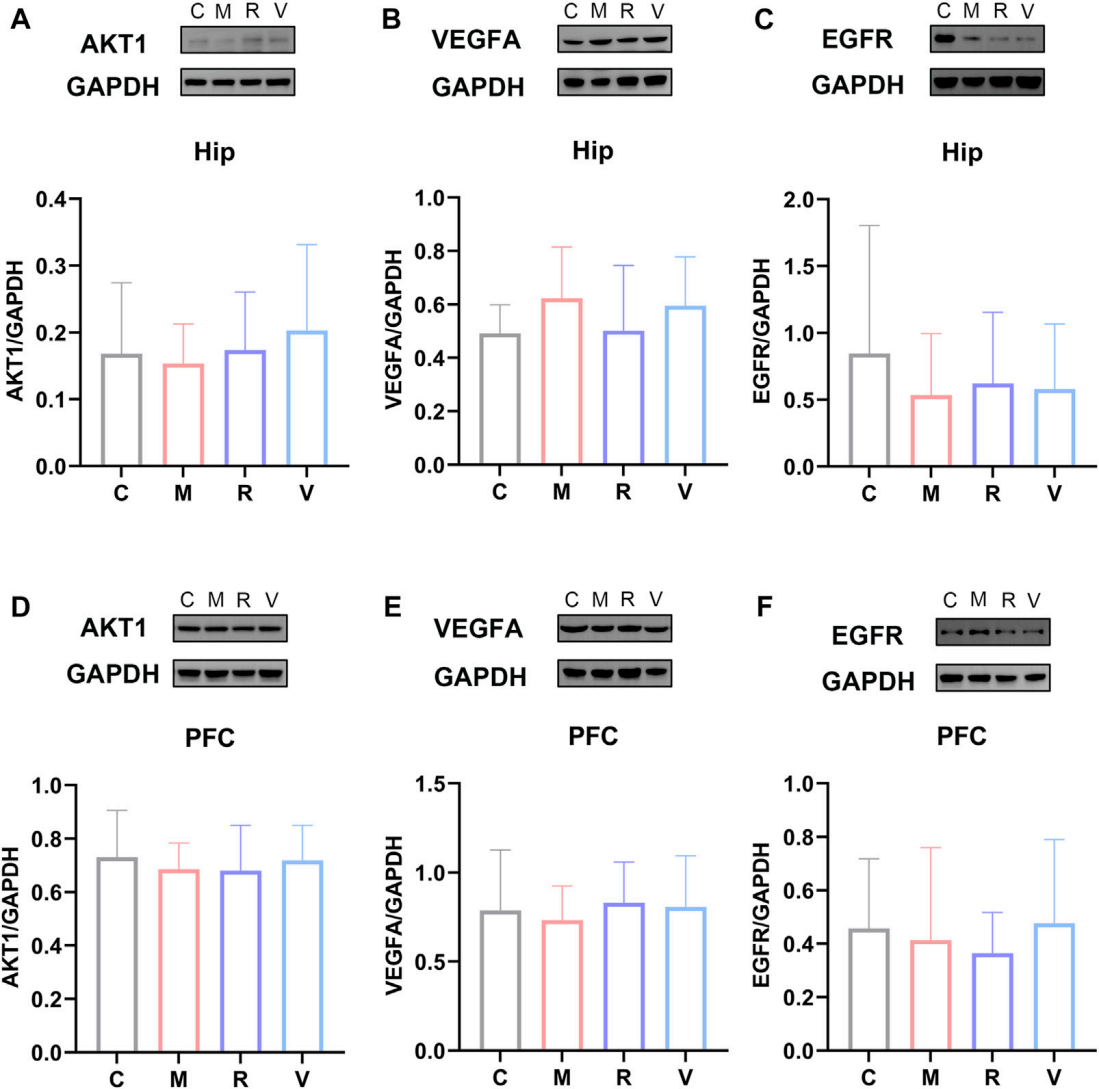
Relative protein expression of AKT1 **(A)**, VEGFA **(B)**, and EGFR **(C)** in the hippocampus. Relative protein abundance of AKT1 **(D)**, VEGFA **(E)** and EGFR **(F)** in the prefrontal cortex. C: Control group, M: CFS group, R: CFS + Rg1 group, and V: CFS+ Oryzanol&VB1 group. Data are mean ± standard deviation, n = 6. *p < 0.05, **p < 0.01 versus Control group; #*p* < 0.05, ##*p* < 0.01 versus CFS group.

We performed western blot analysis to confirm the effect of ginsenoside Rg1 on the protein levels of AKT1, VEGFA and EGFR in the hippocampus and prefrontal cortex. As shown in Figure 10 (A, B, C), ginsenoside Rg1 up-regulates AKT1, EGFR and down-regulates VEGFA protein expression in the hippocampus, despite no statistical significance (*p* > 0.05). In the prefrontal cortex, ginsenoside Rg1 up-regulates VEGFA and down-regulates EGFR protein expression, with no statistical significance (*p* > 0.05) (Figures 10 D, E, F). EGFR protein and mRNA levels were similar in the prefrontal cortex. These results show ginsenoside Rg1 produces anti-fatigue effects by down-regulating EGFR expression.

## 4 Discussion

Chronic fatigue syndrome, also known as myalgic encephalomyelitis, is an underappreciated debilitating disease with a significant impact on patient quality of life (Bornstein et al., 2022). CFS is characterized by chronic, disabling and multi-system disease with no effective treatment methods or diagnostic markers available at present (König et al., 2021; Wirth et al., 2021). CFS is associated with a variety of diseases, such as brucellosis, coronavirus infection, depression, and cancer. Recent studies found that patients recovering from COVID-19 may have persistent debilitating symptoms, and CFS remains the most dominant and common trait in these patients (Bansal et al., 2022). Current treatment guidelines for CFS mainly include cognitive behavioral therapy, graded exercise program and symptomatic therapy (Sapra and Bhandari, 2022), with limited clinical efficacy (Fang et al., 2022). In China, herbal medicine is widely used to treat CFS (Joung et al., 2019; Shin et al., 2021), with a previous meta-analysis showing TCM is safe and effective for CFS treatment (Zhang et al., 2022), which has sparked interest in its application (Wang et al., 2014; Dai et al., 2019; Zhang et al., 2020; Yang et al., 2022).

In this study, we explored the metabolic fingerprints and potential mechanisms of action of ginsenoside Rg1 in CFS treatment. We used multi-factor modeling (load-weighted forced swimming and restriction) to verify the anti-fatigue effects of ginsenoside Rg1 in CFS rats. The forced swimming animal model presents the core symptoms of CFS, such as fatigue and behavioral and cognitive abnormalities, but also pathological changes that have been found in CFS clinical studies (Sarma et al., 2015; Li and Han, 2022). Similarly, chronic restriction is simple to implement and can significantly induce fatigue symptoms (Li and Han, 2022). The rotarod test is widely used to assess athletic endurance with high reproducibility. In fact, the improvement of exercise tolerance is the strongest evidence for the existence of anti-fatigue effects (Kwon et al., 2021). In this study, CFS rats showed a decline in exercise tolerance that could be reversed by ginsenoside Rg1. The Open field test is a classic experimental method that reflects the spontaneous activity behavior of rats in an unfamiliar environment and is used to evaluate the locomotor ability of rats (Ferreira et al., 2022). We showed that the total movement distance in the CFS group was significantly reduced, as illustrated by decreased exercise ability that is also observed in clinical CFS patients (Aerenhouts et al., 2015). D-xylose is a common symptom of spleen deficiency and often used to evaluate intestinal absorption (Zeyue et al., 2022). We found that the D-xylose-excretion rate of CFS rats was statistically different from that of the Control group, indicating that a spleen deficiency syndrome model was successfully established. Accordingly, under similar feeding conditions, CFS mainly affects the gastrointestinal absorption function of rats to induce weight loss. These results demonstrated that the anti-fatigue effect of ginsenoside Rg1 was related to exercise tolerance, spontaneous activity and intestinal absorption function in CFS rats.

We identified 34 differential metabolites in the LC-MS analysis of non-targeted serum metabolites. These metabolites are involved in Taurine and hypotaurine metabolism, Arginine biosynthesis, Ether lipid metabolism, Alanine, aspartate and glutamate metabolism, and Pantothenate and CoA biosynthesis. In addition, we have found that Taurine and Mannose 6-phosphate are key metabolic markers of ginsenoside Rg1 in the treatment of CFS. Previous results showed that the differential metabolites identified here were also associated with CFS in different contexts and animal models, which attests the validity of our work. Among these metabolites, Taurine plays a specifically important role (Germain et al., 2017; Glass et al., 2023). Germain et al. found that CFS patients had low Taurine content, which was mainly affected by lipid metabolism and amino acids (Germain et al., 2017). Taurine is an internal metabolite that acts as an antioxidant and anti-fatigue agent (Schaffer and Kim, 2018), and represents the second most abundant amino acid in human muscle after glutamic acid (Ripps and Shen, 2012). Corsetti et al. suggested that Taurine content is a useful indicator of muscle injury and possible long-term fatigue (Corsetti et al., 2016), as documented by other studies (Luckose et al., 2015; Khalil et al., 2018; Thirupathi et al., 2018; Vidot et al., 2018). Yatabe et al. found that the use of Taurine significantly increased the duration of running time to exhaustion in rats (Yatabe et al., 2009). Moreover, a systematic review showed low-dose Taurine (0.05 g) can reduce muscle fatigue, which suggests it may be an effective drug, especially in high-intensity activities (Chen et al., 2021). Interestingly, Taurine used as a nutritional supplement promotes recovery from muscle injury caused by vigorous exercise (Wei et al., 2021). In addition, Liu et al. found that the therapeutic effect of ginseng on spleen deficiency rats may be realized by regulating Taurine and hypotaurine metabolism, and its metabolites can be used as potential biomarkers for the diagnosis and monitoring of spleen deficiency (Liu et al., 2022). Armstrong et al. analyzed the metabolic profile of the blood and urine of CFS patients and found that CFS patients had abnormal metabolism of alanine, aspartate, glutamate and other energy (Armstrong et al., 2015). These findings confirm the reliability of our results.

Network analysis showed that AKT1, VEGFA and EGFR were the key targets of ginsenoside Rg1 for enabling anti-fatigue effects. AKT1 encodes serine/threonine kinases that play an important role in several normal and pathological cell processes (Millischer et al., 2020), and a deficiency in AKT1 increases energy consumption (Wan et al., 2012). A randomized controlled trial found that AKT1 is an important core target for the treatment of cancer-induced fatigue (Cui et al., 2022). Furthermore, Liu et al. found that ginsenosides could enhance mice body ability and play an anti-fatigue role by upregulating the expression of AKT1 (Liu et al., 2019). We present similar results here and showed ginsenoside Rg1 up-regulates AKT1 protein expression despite not significantly. VEGFA is a key regulator of vascular growth. Angiogenesis in skeletal muscle can maintain oxygen and nutrient supply and clear metabolic byproducts that may lead to fatigue (Mohamad Shalan et al., 2016), which is associated with decreased levels of VEGFA (Wågström et al., 2021). A study on metabolic gene polymorphisms conducted progressive fatigue tests on athletes and identified VEGFA rs2010963C as an “endurance allele” of elite athletes (Ahmetov et al., 2009). We showed that CFS rats have decreased VEGFA levels in the prefrontal cortex, similar to patients with chronic fatigue (Landi et al., 2016).

The pathogenesis of CFS is closely related to the central nervous system (Mohamad Shalan et al., 2016; Sapra and Bhandari, 2022), with this syndrome being included in the neurology category (8E49) in ICD-11 (Gandasegui et al., 2021). In fatigue tests, reduced hippocampus activation is associated with HPA axis dysfunction and higher fatigue ratings (Klaassen et al., 2013). Saury JM et al. believes that the hippocampus plays an important role in the pathogenesis of CFS, and the trigger factors of CFS will also affect the hippocampus, leading to neurocognitive defects and disorders in the regulation of the pressure system and pain perception, which will further affect the hippocampus and trigger a vicious cycle of increased disability (Saury, 2016). There is a negative correlation between fatigue and hippocampus volume in CFS patients (Thapaliya et al., 2022). In addition, the prefrontal cortex has been identified as critical to fatigue (Ifuku et al., 2014). Bilateral prefrontal cortex volume was reduced in CFS patients, and the level of volume reduction in the right prefrontal cortex was correlated with the severity of fatigue (Nakatomi et al., 2014). An fMRI study found abnormal signals of functional activity in the prefrontal cortex of CFS patients (Caseras et al., 2006). These findings all support a strong link between the hippocampus, the prefrontal cortex, and CFS. We further performed PCR and Western Blot analyses on three key targets on the hippocampus and prefrontal cortex of CFS rats. Our results suggest that ginsenoside Rg1 can down-regulate the EGFR protein and mRNA expression. These observations have far-reaching significance for exploring the anti-fatigue mechanism of ginsenoside Rg1 at the molecular level. EGFR is an epidermal growth factor receptor and membrane surface receptor with tyrosine kinase activity (Cheng et al., 2022). Elevated levels of circulating ligands of EGFR, such as epidermal growth factor (EGF) and transforming growth factor α(TGF-α), can inhibit neural signals that drive normal behavior (Rich et al., 2017). Moreover, EGFR activation can stimulate the production of reactive oxygen species (ROS) by stimulating the PI3K pathway (Mansour et al., 2021). Previous studies showed ROS-induced mitochondrial function decline is related to fatigue (Nicolson, 2007; Pieczenik and Neustadt, 2007), and long-term oxidative stress can trigger CFS (Morris et al., 2017). At the same time, the level of oxidative stress in CFS patients is increased and correlated with clinical symptoms (Kennedy et al., 2005). Excessive ROS triggers oxidative stress, which is positively correlated with the severity of CFS symptoms (Gupta et al., 2010). Recent studies showed serum samples from CFS patients induce the production of ROS and nitric oxide in human HMC3 microglia (Gottschalk et al., 2022). A randomized controlled trial found that ginseng can reduce ROS levels in chronic fatigue patients and play an anti-fatigue role (Kim et al., 2013), which corroborates our observations.

## 5 Conclusion

This study explored for the first time the pharmacological mechanisms of ginsenoside Rg1 in CFS treatment using a comprehensive metabolomic model, network analysis and biological methods. We systematically elucidated the multi-target and multi-mechanisms behind ginsenoside Rg1 anti-fatigue effects. Ginsenoside Rg1 can effectively treat CFS by regulating expression levels of AKT1, VEGFA and EGFR, and interfering with the metabolism of Taurine and Mannose 6-phosphate. We experimentally confirmed that EGFR is the most critical target. These findings provide a theoretical basis for the clinical application of ginsenoside Rg1 for CFS treatment.

## Data Availability

The raw data supporting the conclusions of this article will be made available by the authors, without undue reservation.
